# Black Health in Canada: Protocol for a Scoping Review

**DOI:** 10.2196/42212

**Published:** 2023-05-25

**Authors:** Adedoyin Olanlesi-Aliu, Dominic Alaazi, Bukola Salami

**Affiliations:** 1 Faculty of Nursing University of Alberta Edmonton, AB Canada

**Keywords:** Black people, inequities, mental health, reproductive and sexual health, HIV, social determinants of health, Canada

## Abstract

**Background:**

Black Canadians experience poor health care, poor health outcomes, and a greater burden of health inequalities, much of which is rooted in the unequal distribution of social determinants of health. Despite Canada’s emphasis on social inclusion, Canada’s Black population faces substantial social inequities that affect their health and well-being. These disparities may specifically be attributed to racial discrimination, immigration status, precarious housing, underemployment, and increased poverty among Black Canadians.

**Objective:**

This paper describes a protocol for a scoping review that aims to understand the range and nature of research conducted on the health of Black Canadians as well as the gaps in this literature.

**Methods:**

Arksey and O'Malley’s methodological framework guided the conduct of the scoping review. We searched electronic databases (CINAHL, Embase, Global Health, MEDLINE, PsycINFO, Scopus, Sociological Abstracts, and Web of Science) and grey literature sources for peer-reviewed articles and grey reports on the health of Black Canadians. Six reviewers independently screened the abstracts and full text of studies to determine eligibility for inclusion. According to the PRISMA-ScR (Preferred Reporting Items for Systematics Reviews and Meta-Analyses extension for Scoping Reviews) guidelines, the findings will be synthesized quantitatively and qualitatively through thematic analysis.

**Results:**

Title, abstract, and full-text screening concluded in October 2022. Data collection is in progress and is expected to be completed by April 2023. Data analysis and drafting of the manuscript will be done thereafter. Findings from the scoping review are expected to be provided for peer review in 2023.

**Conclusions:**

This review will collect important data and evidence related to the health (mental, reproductive, and sexual; social determinants of health) of the Black population in Canada. The findings could help identify existing gaps in the health of Black individuals in Canada and inform future research paradigms. The findings will further inform the development of a knowledge hub on Black Canadians’ health.

**International Registered Report Identifier (IRRID):**

PRR1-10.2196/42212

## Introduction

### Background

Canada is a racially and culturally diverse country, much of which has been fueled by immigration and settlement of racial minority groups from Asia, the Caribbean, and Africa since the 1960s [1,2]. Today, Canada is one of the most popular immigrant destinations globally, with a growing inflow of Black migrants from sub-Saharan African and Caribbean nations [3]. Black people are the third-leading minority group in Canada, nearing 3.5% of the country’s total population, with a projected increase to 5.6% by 2036 [4]. Despite persistent attempts to support multiculturalism and racial equality in Canada, race-based social and health inequalities are still widespread [5,6]. The social, economic, and political conditions in which people grow, live, and work are largely responsible for the observed inequalities [4,7,8]. For this scoping review, we will adopt the World Health Organization’s definition of health: “Health is a state of complete physical, mental and social well-being and not merely the absence of disease or infirmity” [9]. Therefore, health is broadly defined to include disease conditions; physical, mental, emotional, and spiritual well-being; access to and utilization of health care; barriers and facilitators to health; health interventions, etc.

Black people in Canada are disproportionately affected by social and health inequalities, including poor access to quality health care, education, employment, housing, and mental health support [10-13]. The inequities produced by these social determinants of health are exacerbated by Black people’s experiences of precarious immigration status, poverty, and racial discrimination at both interpersonal and institutional levels [4,8,14,15]. Yet, Black people aged 25 to 54 years are more likely to have a bachelor’s degree or higher (41.1%) than their nonvisible minority or non-Indigenous counterparts (34.2%) [16]. In 2018, the proportion of the Black population in Canada who resided in rented dwellings (n=695,900, 52%) was twice that for the overall population (n=9,749,700, 27%); furthermore, of those who lived in rented dwellings, the proportion of Black individuals living in unsuitable housing (n=209,800, 30%) was notably higher than that of the overall population (n=1,808,900, 19) [17].

The prolonged exposure to trauma caused by oppression, colonialism, racism, and segregation among Black people in Canada has resulted in them being underprivileged with respect to access to health services and social support systems [13]. Black Canadians experience disparities in health outcomes when compared to other ethno-racial groups in Canada [18]. For instance, heart disease and stroke are among the prominent causes of death in Canada, and Black populations are among those with the highest rate of risk factors for these conditions [4]. In addition, the proportion of Black Canadians aged 18 years and older who rated their health status to be either fair or poor was higher (14.2%) than that of their White Canadian counterparts (11.3%) [4]. About 64% of young Black women between the ages of 12 and 17 years described their mental health to be “excellent” or “very good,” which is substantially lower than the 77.2% reported by their White counterparts [4]. Black Canadians experience notable social and economic difficulties that have deleterious consequences on their mental health [19]. This includes disparities in access to mental health services among Black Canadians, which may be attributed to having few Black mental health service providers in the health care system. Indeed, Black Canadians have indicated their interest in accessing mental health care services with Black service providers [20]. The mental health of youth and children in Canada has become a public health problem and has been attributed to the inaccessibility of mental health care services [21]. The Black community in particular has emphasized the need to tackle the mental health issues that are prevalent among youth and children in their communities [22]. Finally, the Black population in Canada has been disproportionately burdened with HIV in the last 20 years. In 2017, a total of 25% of reported HIV cases were among the Black population, which is noteworthy as Black people only account for 3.5% of Canada’s total population; in contrast, their White counterparts, who make up almost 75% of Canada’s population, represent only 35% of reported cases [23]. Timely access and provision of health care services are major determinants of health for people living with HIV; however, Black populations face inequities that hinder their access to health care services [24].

These disparities suggest a need for innovative research focusing on Black Canadians’ health. A scoping review of the literature chronicling existing research on the health of Black Canadians is a critical step in this regard. This project will synthesize this literature and explain the nature and breadth of research on the health of Black Canadians, as well as gaps to be addressed by future research. The findings from the scoping review will inform the creation of a knowledge hub on Black health in Canada. The purpose of this scoping review is thus to map out the scope of the literature on health (including disease conditions; physical, mental, emotional, and spiritual well-being; access to and utilization of health care; barriers and facilitators to health; health interventions, etc).

### Objectives

The review will address two specific objectives:

1. To identify the extent, range, and nature of the literature on the health of Black Canadians; and
2. To identify gaps in the literature on the health of Black Canadians.

## Methods

### Stage 1: Identifying the Research Questions

Arksey and O’Malley [25] proposed an iterative process for developing research questions, where each modification is based on intensifying familiarity with the literature. Thus, the research question was as follows: What is known in the existing literature about the health of Black individuals in Canada?

### Stage 2: Identifying Relevant Studies

Bibliographic database searches were conducted in June 2022. At this stage, we developed a search strategy, including the keywords to use, and searched the following databases: CINAHL, Embase, Global Health, MEDLINE, PsycINFO, Scopus, Sociological Abstracts, and Web of Science (Multimedia Appendix 1). We completed a search for grey literature by searching Google and the websites of agencies.

### Stage 3: Study Selection

#### Search Strategy

**Figure 1:**
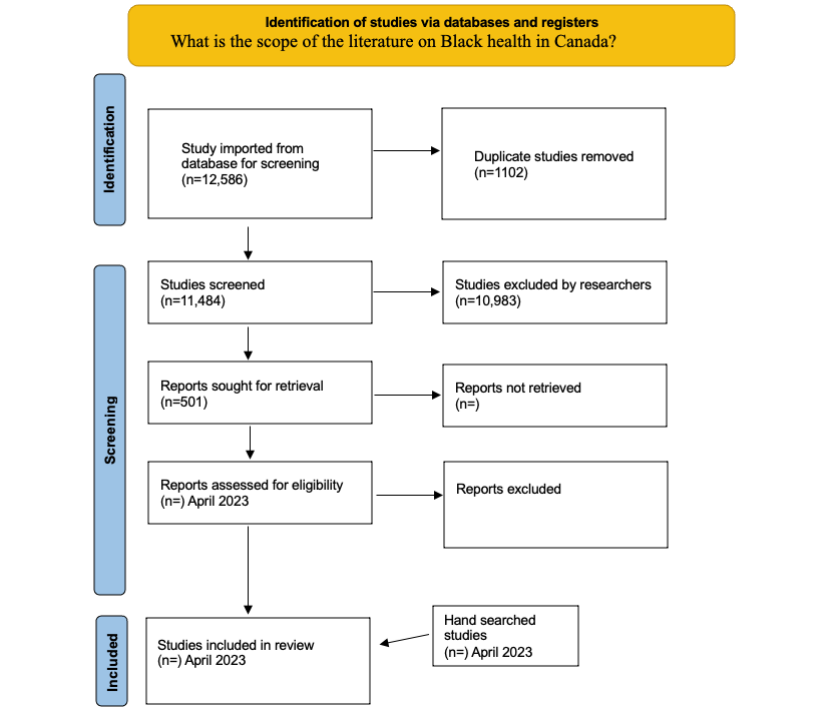
PRISMA (Preferred Reporting Items for Systematic reviews and Meta-Analyses) 2020 flow diagram: Black health in Canada. Adapted from Page et al [26].

A total of 12,586 articles were retrieved, of which 1102 were duplicates. A total of 11,484 citations were downloaded from the bibliographic databases into an Endnote library (Endnote 20) and, from there, exported into Covidence where titles and abstracts were reviewed independently by 2 research assistants. Studies that did not meet the inclusion criteria (n=10,983) were excluded. The full text versions of the remaining 501 articles have been retrieved and are being independently reviewed by 2 research assistants to determine the eligibility of the articles. A third researcher will resolve disagreements between the 2 research assistants at all review stages. Reference lists of included articles will be scanned for any additional articles that meet the study’s inclusion criteria (Figure 1).

#### Inclusion Criteria

##### Participants

The participants will include any Black/African individuals, populations, and communities living in Canada. We define Black Canadians as “People of African descent and those who define themselves as such, who are now residents/citizens of Canada” [27].

##### Concept

For this scoping review, we define health as “a state of complete physical, mental, and social well-being and not merely the absence of disease or infirmity” [9]. A broader range of languages will be considered, and language translators will be engaged with articles published in other languages. The review team will include Black Canadians to ensure that the review is culturally sensitive and inclusive.

##### Geographical Location

Studies conducted in Canada will be included.

##### Types of Sources

Eligible study designs will include qualitative, quantitative, and mixed methods study designs. In addition, unpublished theses and dissertations will be included in this scoping review.

#### Exclusion Criteria

Exclusion criteria will include the following:

- Study types: secondary literature (scoping reviews, literature reviews, systematic reviews, and meta-analysis), letters to the editor, protocols, and case series
- Concept: studies that did not target the health of Black Canadians

### Stage 4: Charting the Data

A data extraction sheet will be developed and used for extraction (Multimedia Appendix 2). It is anticipated that the data categories will include standard information such as author name(s), year of publication, the purpose of the study, study population, methods, results/findings, and comments/implications. Two trained research assistants will independently read each article and extract data. A third reviewer will verify a sample of the extraction for completeness and accuracy. We will ensure that methodological quality checks are conducted to ensure the consistency, accuracy, and thoroughness of the information extracted.

### Stage 5: Collating, Summarizing, and Reporting the Results

Quantitative data will be analyzed through a numerical summary. Qualitative studies will be analyzed using thematic analysis to synthesize and categorize the findings of the included studies into themes (drawing from Braun and Clarke [28]). To do so, we will read the included articles several times and familiarize ourselves with the data. Subsequently, we will compile codes into potential themes, group all data relevant to the potential theme, and compare data across the coded excerpts and the entire data set.

## Results

We started this project in June 2022 with the development of the search strategy and literature search. As of October 2022, the study team has completed the title, abstract, and full-text screening of imported citations. The articles selected to be represented in the table are important because they meet the inclusion criteria and contribute to the understanding of the health, quality of life, and well-being of Black Canadians. Articles that survive the screening process will provide valuable evidence on the topic of interest. As the study team is expected to complete data collection by April 2023 and the analysis of data and drafting of manuscripts will be done thereafter, this study may provide timely and relevant information to inform the development of the knowledge hub on the health of Black Canadians.

## Discussion

### Expected Findings

This research protocol aims to guide the researchers in conducting research using a method that entails exploring and mapping literature and identifying research gaps in the health of Black Canadians.

Several study protocols or reviews have been published on the health of Black Canadians. However, these tend to focus too narrowly on specific aspects of health and often neglect a more comprehensive picture. Nguemo et al [29] performed a scoping review of substance use disorders among Black Canadians and found that low income, economic deprivation, unemployment, the lack of health education, immigration, and limited access to treatment services intensified the rate of substance use among Black Canadians. Jefferies et al [30] described food security in African Canadian communities and indicated that food access and food availability are associated with an increase in the rate of dietary acculturation. Similarly, Lofters et al [31] noted that findings from a future review would help in the implementation of human papillomavirus self-sampling and the improvement of uptake of cervical cancer screening among women from African countries who resides in Ontario. Finally, Dogba et al [32] published a scoping review protocol to identify obstacles and enablers to participation by diabetic ethnocultural minority immigrants in retinopathy screening as well as generate results that would help in developing intervention schemes for behavioral modification.

The scoping review will evaluate various disease prevention strategies, access to and utilization of health care, and facilitators to health in Black individuals and communities in Canada. We will explore the resistance of Black Canadians in relation to the barriers they face in accessing health care services and other health intervention programs. Additionally, the impacts of social determinants of health and intersections of dimensions such as gender identity, sexual orientation, age, disabilities, language, educational attainment, and immigration status on the health of Black Canadians will be evaluated.

The completed scoping review will inform research, programs, and services as well as identify potential areas to facilitate and improve the health and well-being of Black Canadians. The findings will be relevant to a variety of audiences, such as researchers, policy makers, stakeholders, health care providers, clinicians, and governments who strive to better understand the health, quality of life, social determinants of health, and well-being of Black Canadians.

### Limitations

One limitation of this scooping review is the wide range of inclusion criteria. Therefore, the hand searching of the literature by the research team may result in individual interpretation of the criteria. The lack of critical appraisal and the chance of omitting relevant studies have been reported as major challenges in conducting scoping reviews [33,34]. We acknowledge some biases such as the possibility of omitting studies not captured by our search terms or databases. As there is potential disagreement among reviewers in determining the eligibility of studies, a third reviewer will resolve disagreements at all review stages.

### Implications

Our review will address the absence of a comprehensive review on the health of Black Canadians by taking a broader view of health than previous efforts. We will identify the full range of research on the health of Black Canadians and categorize such work into themes reflecting specific topical areas of health and well-being. We will synthesize the literature and identify trends and knowledge gaps in each category of research articles. This effort will contribute to the development of a knowledge hub on the health of Black Canadians that will serve as a resource for the Black communities, policy makers, stakeholders, health care providers, and the government at large. Our findings will also be disseminated in peer-reviewed manuscripts and at conferences.

## Appendix

Multimedia Appendix 1 Search strategy for Black people’s health in Canada.

Multimedia Appendix 2 Data extraction instrument.

## Abbreviations

PRISMA-ScR: Preferred Reporting Items for Systematics Reviews and Meta-Analyses extension for Scoping Reviews
