# Supplementary material for: Black Health in Canada: Protocol for a Scoping Review
Source: JMIR Res Protoc. 2023 May 25;12:e42212. doi: 10.2196/42212 (PMC10251231; doi:10.2196/42212)
Supplement: Multimedia Appendix 1 [file resprot_v12i1e42212_app1.docx]

**Multimedia Appendix 1. Search Strategy for Black people’s health in Canada**

| **CINAHL Plus with Full Text via EBSCOhost** | |  |
| --- | --- | --- |
| **Last searched: June 29, 2022** | |  |
| LIMITERS:   - None | |  |
| **#** | **Query** | **Results** |
| S1 | (MH “Black Persons”) | 58055 |
| S2 | (black* or african* or caribbean or afro* or "person of colo#r" or "people of colo#r" or colo#red or "dark-skin*" or BIPOC or ((racial or ethnic) N2 minorit*)) | 150577 |
| S3 | (MH "Africa+") OR (MH "Africa, Northern+") OR (MH "Africa South of the Sahara+") OR (MH "Africa, Central+") OR (MH "Africa, Eastern+") OR (MH "Africa, Southern+") OR (MH "Africa, Western+") | 95606 |
| S4 | TI ( (algeria* or angola* or benin* or Botswana* or "burkina faso" or Burundi or Cameroon or "Cape Verde" or "Central African Republic" or chad or Comoros or Congo* or "cote d'ivoire" or "Ivory Coast" or Djibouti or Egypt* or Guinea* or Eritrea or Ethiopia* or Gabon or Gambia* or ghana* or Kenya* or Lesotho or Liberia* or Libya* or Madagascar or Malawi* or mali* or Mauritania* or Mauritius or Morocco or Mozambique or Namibia* or niger or nigeria* or rwanda* or "sao tome and principe" or senegal* or seychelles or "sierra leone" or somalia* or "south africa" or sudan or swaziland or tanzania* or togo or Tunisia* or uganda* or Zambia* or Zimbabwe*) ) OR AB ( (algeria* or angola* or benin* or Botswana* or "burkina faso" or Burundi or Cameroon or "Cape Verde" or "Central African Republic" or chad or Comoros or Congo* or "cote d'ivoire" or "Ivory Coast" or Djibouti or Egypt* or Guinea* or Eritrea or Ethiopia* or Gabon or Gambia* or ghana* or Kenya* or Lesotho or Liberia* or Libya* or Madagascar or Malawi* or mali* or Mauritania* or Mauritius or Morocco or Mozambique or Namibia* or niger or nigeria* or rwanda* or "sao tome and principe" or senegal* or seychelles or "sierra leone" or somalia* or "south africa" or sudan or swaziland or tanzania* or togo or Tunisia* or uganda* or Zambia* or Zimbabwe*) ) | 170726 |
| S5 | (MH "West Indies+") | 11244 |
| S6 | (Cuba* or "Dominican Republic" or Haiti* or Hispaniola* or "Puerto Rico" or "Puerto Rican*" or Jamaica* or Barbados or Dominica or Grenad* or "Saint Lucia" or trinidad* or Bahama* or "Virgin Islands" or Anguilla or "Saint Kitts" or Antigua or "turks and Caicos" or "West Indies" or "Saint Vincent") | 15821 |
| S7 | S1 OR S2 OR S3 OR S4 OR S5 OR S6 | 347786 |
| S8 | MH (“Health”) OR MH (“Wellness”) OR MH (“Health Status”) | 89794 |
| S9 | TI (health OR wellbeing OR “well being” OR well-being OR wellness OR well-ness OR sick* OR illness* OR disease* OR disorder* OR infection* OR morbidit* OR mortalit*) OR AB (health OR wellbeing OR “well being” OR well-being OR wellness OR well-ness OR sick* OR illness* OR disease* OR disorder* OR infection* OR morbidit* OR mortalit*) OR **AB…** | 2377880 |
| S10 | S8 OR S9 | 2399992 |
| S11 | MH (“Canada+”) | 110506 |
| S12 | TI (Canad* OR "British Columbia" OR "Colombie Britannique" OR Alberta* OR Saskatchewan OR Manitoba* OR Ontario OR Quebec OR "Nouveau Brunswick" OR "Nova Scotia" OR "Nouvelle Ecosse" OR "Prince Edward Island" OR Newfoundland OR Labrador OR Nunavut OR NWT OR "northwest territories" OR Yukon OR Nunavik OR Inuvialuit) OR AB (Canad* OR "British Columbia" OR "Colombie Britannique" OR Alberta* OR Saskatchewan OR Manitoba* OR Ontario OR Quebec OR "Nouveau Brunswick" OR "Nova Scotia" OR "Nouvelle Ecosse" OR "Prince Edward Island" OR Newfoundland OR Labrador OR Nunavut OR NWT OR "northwest territories" OR Yukon OR Nunavik OR Inuvialuit) OR **AB…** | 159537 |
| S13 | S11 OR S12 |  |
| S14 | S7 AND S10 AND S13 | 3031 |

| **Scopus** | |  |
| --- | --- | --- |
| **Last searched: June 29, 2022** | |  |
| LIMITERS:   - None | |  |
| **#** | **Query** | **Results** |
|  | TITLE-ABS-KEY (( black* OR african* OR caribbean OR afro* OR "person of colo?r" OR "people of colo?r" OR colo?red OR "dark-skin*" OR bipoc OR ( ( racial OR ethnic ) W/2 minorit* ) AND (health OR wellbeing OR “well being” OR well-being OR wellness OR well-ness OR sick* OR illness* OR disease* OR disorder* OR infection* OR morbidit* OR mortalit*) AND (Canad* OR "British Columbia" OR "Colombie Britannique" OR Alberta* OR Saskatchewan OR Manitoba* OR Ontario OR Quebec OR "Nouveau Brunswick" OR "Nova Scotia" OR "Nouvelle Ecosse" OR "Prince Edward Island" OR Newfoundland OR Labrador OR Nunavut OR NWT OR "northwest territories" OR Yukon OR Nunavik OR Inuvialuit)) | 4918 |

| EMBASE | |  |
| --- | --- | --- |
| **Last searched: June 29, 2022** | |  |
| LIMITERS:   - None | |  |
| **#** | **Query** | **Results** |
| 1 | exp black person/ | 129256 |
| 2 | (black* or african* or caribbean or afro* or "person of colo?r" or "people of colo?r" or colo?red or "dark-skin*" or BIPOC or ((racial or ethnic) adj2 minorit*)).mp. | 621514 |
| 3 | 1 or 2 | 621514 |
| 4 | Health/ or exp mental health/ | 411297 |
| 5 | exp Canada/ or (Canad* OR "British Columbia" OR "Colombie Britannique" OR Alberta* OR Saskatchewan OR Manitoba* OR Ontario OR Quebec OR "Nouveau Brunswick" OR "New Brunswick" OR "Nova Scotia" OR "Nouvelle Ecosse" OR "Prince Edward Island" OR Newfoundland OR Labrador OR Nunavut OR NWT OR "Northwest Territories" OR Yukon OR Nunavik OR Inuvialuit) | 354163 |
| 6 | 3 and 4 and 5 | 333 |

| GLOBAL HEALTH | |  |
| --- | --- | --- |
| **Last searched: June 29, 2022** | |  |
| LIMITERS:   - None | |  |
| **#** | **Query** | **Results** |
| 1 | black people/ | 20329 |
| 2 | (black* or african* or caribbean or afro* or "person of colo?r" or "people of colo?r" or colo?red or "dark-skin*" or racial* or race? or ((racial or ethnic) adj2 minorit*)).mp. | 224610 |
| 3 | 1 or 2 | 224610 |
| 4 | (health*or wellness* or health status*).mp. [mp=abstract, title, original title, heading words, cabicodes words] | 20162 |
| 5 | exp Canada/ or (Canad* OR "British Columbia" OR "Colombie Britannique" OR Alberta* OR Saskatchewan OR Manitoba* OR Ontario OR Quebec OR "Nouveau Brunswick" OR "New Brunswick" OR "Nova Scotia" OR "Nouvelle Ecosse" OR "Prince Edward Island" OR Newfoundland OR Labrador OR Nunavut OR NWT OR "Northwest Territories" OR Yukon OR Nunavik OR Inuvialuit) .mp. [mp=abstract, title, original title, heading words, cabicodes words] | 63837 |
| 6 | 3 and 4 and 5 | 36 |

| PsycInfo | |  |
| --- | --- | --- |
| **Last searched: June 20, 2022** | |  |
| LIMITERS:   - None | |  |
| **#** | **Query** | **Results** |
| 1 | african cultural groups/ | 3255 |
| 2 | blacks/ | 56599 |
| 3 | (black* or african* or caribbean or afro* or "person of colo?r" or "people of colo?r" or colo?red or "dark-skin*" or BIPOC or ((racial or ethnic) adj2 minorit*)).mp. | 170499 |
| 4 | 1 or 2 or 3 | 170499 |
| 5 | (health*or wellness* or health status*).mp. [mp=abstract, title, original title, heading word, table of contents, key concepts, original title, tests & measures, mesh word] | 1016612 |
| 6 | (Canad* or "British Columbia" or "Colombie Britannique" or Alberta* or Saskatchewan or Manitoba* or Ontario or Quebec or "Nouveau Brunswick" or "New Brunswick" or "Nova Scotia" or "Nouvelle Ecosse" or "Prince Edward Island" or Newfoundland or Labrador or Nunavut or NWT or "Northwest Territories" or Yukon or Nunavik or Inuvialuit).mp. [mp=abstract, title, original title, heading word, table of contents, key concepts, original title, tests & measures, mesh word] | 68981 |
| 7 | 4 and 5 and 6 | 624 |

| MEDLINE | |  |
| --- | --- | --- |
| **Last searched: June 29, 2022** | |  |
| LIMITERS:   - None | |  |
| **#** | **Query** | **Results** |
| 1 | exp Blacks/ | 94890 |
| 2 | (black* or african* or caribbean or afro* or "person of colo?r" or "people of colo?r" or colo?red or "dark-skin*" or BIPOC or ((racial or ethnic) adj2 minorit*)) | 381403 |
| 3 | 1 or 2 | 406131 |
| 4 | exp Health/ | 417741 |
| 5 | (health or wellbeing or wellness or disease* or disorder* or infection* or morbidit* or mortalit*) | 8370564 |
| 6 | 4 or 5 | 8512600 |
| 7 | exp Canada/ | 175994 |
| 8 | (Canad* OR "British Columbia" OR "Colombie Britannique" OR Alberta* OR Saskatchewan OR Manitoba* OR Ontario OR Quebec OR "Nouveau Brunswick" OR "New Brunswick" OR "Nova Scotia" OR "Nouvelle Ecosse" OR "Prince Edward Island" OR Newfoundland OR Labrador OR Nunavut OR NWT OR "Northwest Territories" OR Yukon OR Nunavik OR Inuvialuit) | 189805 |
| 9 | 7 or 8 | 261603 |
| 10 | 3 and 6 and 9 | 2441 |

| Web of Science | |  |
| --- | --- | --- |
| **Last searched: June 30, 2022** | |  |
| LIMITERS:   - None | |  |
| **#** | **Query** | **Results** |
| **1** | TI=(black* or african* or caribbean or afro* or "person of colo?r" or "people of colo?r" or colo?red or "dark-skin*" or BIPOC or ((racial or ethnic) adj2 minorit*)) | 779262 |
| **2** | AB=(black* or african* or caribbean or afro* or "person of colo?r" or "people of colo?r" or colo?red or "dark-skin*" or BIPOC or ((racial or ethnic) adj2 minorit*)) | 1510787 |
| **3** | 1 OR 2 | 1825317 |
| **4** | **TI=**(health or wellbeing or wellness or disease* or disorder* or infection* or morbidit* or mortalit*) | 45614605 |
| **5** | **AB=**(health or wellbeing or wellness or disease* or disorder* or infection* or morbidit* or mortalit*) | 11926288 |
| **6** | 4 OR 5 | 14365917 |
| **7** | **TI=**(Canad* OR "British Columbia" OR "Colombie Britannique" OR Alberta* OR Saskatchewan OR Manitoba* OR Ontario OR Quebec OR "Nouveau Brunswick" OR "New Brunswick" OR "Nova Scotia" OR "Nouvelle Ecosse" OR "Prince Edward Island" OR Newfoundland OR Labrador OR Nunavut OR NWT OR "Northwest Territories" OR Yukon OR Nunavik OR Inuvialuit) | 370489 |
| **8** | **AB=**(Canad* OR "British Columbia" OR "Colombie Britannique" OR Alberta* OR Saskatchewan OR Manitoba* OR Ontario OR Quebec OR "Nouveau Brunswick" OR "New Brunswick" OR "Nova Scotia" OR "Nouvelle Ecosse" OR "Prince Edward Island" OR Newfoundland OR Labrador OR Nunavut OR NWT OR "Northwest Territories" OR Yukon OR Nunavik OR Inuvialuit) | 485149 |
| **9** | 7 OR 8 | 706747 |
| **10** | 3 AND 6 AND 9 | 1677 |

| Sociological Abstract via proquest | |  |
| --- | --- | --- |
| **Last searched: June 30, 2022** | |  |
| LIMITERS:   - None | |  |
| **#** | **Query** | **Results** |
| **1** | ("black" OR "african" OR "caribbean" OR "afro" OR "person of colo?r" OR "people" OR "colo?r" OR "colo?red" OR "dark-skin" OR "BIPOC" OR "racial" OR "ethnic" OR "minorit") |  |
| **2** | (black* OR african* OR caribbean OR afro* OR "person of colo?r" OR "people of colo?r" OR colo?red OR"dark-skin*" OR BIPOC OR ((racial or ethnic) adj2 minorit*)) |  |
| **3** | SU.EXACT("Health" OR "Health Status" OR "Well Being" OR "Mental Health") |  |
| **4** | NOFT (health OR wellbeing OR “well being” OR well-being OR wellness OR well-ness OR sick* OR illness* OR disease* OR disorder* OR infection* OR morbidit* OR mortalit*) |  |
| **5** | NOFT (Canad* OR "British Columbia" OR "Colombie Britannique" OR Alberta* OR Saskatchewan OR Manitoba* OR Ontario OR Quebec OR "Nouveau Brunswick" OR "Nova Scotia" OR "Nouvelle Ecosse" OR "Prince Edward Island" OR Newfoundland OR Labrador OR Nunavut OR NWT OR "northwest territories" OR Yukon OR Nunavik OR Inuvialuit) | **143** |
