# Supplementary material for: Black Health in Canada: Protocol for a Scoping Review
Source: JMIR Res Protoc. 2023 May 25;12:e42212. doi: 10.2196/42212 (PMC10251231; doi:10.2196/42212)
Supplement: Multimedia Appendix 2 [file resprot_v12i1e42212_app2.docx]

**Multimedia Appendix 2. Data Extraction Instrument.**

| **Author (s)** | **Study Purpose** | **Study population** | **Sample size** | **Methods** | **Province** | **Results** | **Implication** |
| --- | --- | --- | --- | --- | --- | --- | --- |
|  |  |  |  |  |  |  |  |
|  |  |  |  |  |  |  |  |
|  |  |  |  |  |  |  |  |
|  |  |  |  |  |  |  |  |
|  |  |  |  |  |  |  |  |
